# Factors associated with mosquito net use by individuals in households owning nets in Ethiopia

**DOI:** 10.1186/1475-2875-10-354

**Published:** 2011-12-13

**Authors:** Patricia M Graves, Jeremiah M Ngondi, Jimee Hwang, Asefaw Getachew, Teshome Gebre, Aryc W Mosher, Amy E Patterson, Estifanos B Shargie, Zerihun Tadesse, Adam Wolkon, Richard Reithinger, Paul M Emerson, Frank O Richards

**Affiliations:** 1 The Carter Center, Atlanta, GA, USA; 2 University of Cambridge, Cambridge, UK; 3 Centers for Disease Control and Prevention, Atlanta, GA, USA; 4 Global Health Group, University of California, San Francisco, CA, USA; 5 MACEPA, Addis Ababa, Ethiopia; 6 The Carter Center, Addis Ababa, Ethiopia; 7 Strategic Information Team, The Global Fund to Fight AIDS, Tuberculosis and Malaria, Chemin de Blandonnet 8, Geneva, 1214, Vernier, Switzerland; 8 Federal Ministry of Health, Addis Ababa, Ethiopia; 9 US Agency for International Development, Addis Ababa, Ethiopia; 10 School of Public Health, Tropical Medicine and Rehabilitation Sciences, Faculty of Medicine, Health and Molecular Sciences, PO Box 6811, Cairns Qld 4870, Australia; 11 International Trachoma Initiative, The Task Force for Global Health, Ethio-China Friendship Road, Dire Dawa Building, 5th Floor, Room 301, PO Box 10001, Addis Ababa, Ethiopia

**Keywords:** Malaria, Mosquito net, Ethiopia, GLLAMM, Survey, Net use

## Abstract

**Background:**

Ownership of insecticidal mosquito nets has dramatically increased in Ethiopia since 2006, but the proportion of persons with access to such nets who use them has declined. It is important to understand individual level net use factors in the context of the home to modify programmes so as to maximize net use.

**Methods:**

Generalized linear latent and mixed models (GLLAMM) were used to investigate net use using individual level data from people living in net-owning households from two surveys in Ethiopia: baseline 2006 included 12,678 individuals from 2,468 households and a sub-sample of the Malaria Indicator Survey (MIS) in 2007 included 14,663 individuals from 3,353 households. Individual factors (age, sex, pregnancy); net factors (condition, age, net density); household factors (number of rooms [2006] or sleeping spaces [2007], IRS, women's knowledge and school attendance [2007 only], wealth, altitude); and cluster level factors (rural or urban) were investigated in univariate and multi-variable models for each survey.

**Results:**

In 2006, increased net use was associated with: age 25-49 years (adjusted (a) OR = 1.4, 95% confidence interval (CI) 1.2-1.7) compared to children U5; female gender (aOR = 1.4; 95% CI 1.2-1.5); fewer nets with holes (Ptrend = 0.002); and increasing net density (Ptrend < 0.001). Reduced net use was associated with: age 5-24 years (aOR = 0.2; 95% CI 0.2-0.3). In 2007, increased net use was associated with: female gender (aOR = 1.3; 95% CI 1.1-1.6); fewer nets with holes (aOR _[all nets in HH good] _= 1.6; 95% CI 1.2-2.1); increasing net density (Ptrend < 0.001); increased women's malaria knowledge (Ptrend < 0.001); and urban clusters (aOR = 2.5; 95% CI 1.5-4.1). Reduced net use was associated with: age 5-24 years (aOR = 0.3; 95% CI 0.2-0.4); number of sleeping spaces (aOR _[per additional space] _= 0.6, 95% CI 0.5-0.7); more old nets (aOR _[all nets in HH older than 12 months] _= 0.5; 95% CI 0.3-0.7); and increasing household altitude (Ptrend < 0.001).

**Conclusion:**

In both surveys, net use was more likely by women, if nets had fewer holes and were at higher net per person density within households. School-age children and young adults were much less likely to use a net. Increasing availability of nets within households (i.e. increasing net density), and improving net condition while focusing on education and promotion of net use, especially in school-age children and young adults in rural areas, are crucial areas for intervention to ensure maximum net use and consequent reduction of malaria transmission.

## Background

Large donations of free nets have allowed net ownership by households in Africa to increase markedly since 2000 [1], and ownership of long-lasting insecticidal nets (LLIN) in Ethiopia has increased dramatically in the last few years [2,3]. After an initial peak in ownership and use shortly after distribution, net ownership and use have been observed to drop off in several countries [4,5].

Clearly, net ownership is a necessary prerequisite for net use. However, whether or not a net owner will use a net every night, some nights, or not at all depends on complex multi-level interactions between individual characteristics, household characteristics, social and cultural factors, community-level factors, aspects of the physical environment and characteristics of the net itself.

In Ethiopia, a decline was observed in net use in households owning nets between two representative household surveys conducted approximately one year apart, and this did not appear to be associated with differences in sampling or any climatic or seasonal differences between the survey years [3]. To investigate further the reasons for the decline, characteristics of nets that may be impacting their use in Ethiopia were studied. Between 2006 and 2007, the proportion of households owning at least one net increased dramatically from 19.6% to 65.6%, but the proportion of nets used the previous night in households owning nets decreased from 85.1% to 56.0% [3,6]. In the net level analysis, factors independently associated in both surveys with reduced likelihood that a net would be used were: increasing net age, increasing damage of nets, increasing household net density (nets/person), and increasing altitude (> 2,000 m). Factors associated with increased likelihood of a net being used were: increasing wealth index (at both surveys), LLIN net type (in 2006), and household status of indoor residual spraying with insecticide (in 2007) [6].

At the individual level, factors influencing net use have been reported to include age and gender [5,7-10], education, occupation/livelihood [11-13], degree of control over household decision-making [14], malaria knowledge, beliefs and risk perceptions [5,15-21], perceived benefits and disadvantages of nets [5,17,18,21], trust in health workers providing health education and LLINs [22], knowledge of appropriate net use/care practices, and net-hanging skills [23-25]. Household level determinants of net use include household size and composition [14], the number of children under five years of age (U5) in the household, intra-household sleeping arrangements [5,13,26], household structure and space [5,7,9,18,26,27], household decision-making processes and power structures [14], and use of other vector control measures [14,19,28]. At the community level, social norms and values [13], cultural beliefs and practices [13,20], mechanisms of LLIN distribution and distance to LLIN suppliers [14,20], rumours about LLINs [20] and social support and pressure [24] all have the potential to influence net use by individuals and within households. For example, white nets may be associated with burial shrouds and death, and free nets may be regarded as toxic or even deliberately harmful to recipient groups [20]. Important environmental factors include climate and temperature [7,18], perceived mosquito density [19], availability and proximity of land for farming and grazing livestock [13]. Characteristics of the nets themselves, such as their cost, size, shape, colour, physical condition, type of insecticide used and perceived durability have also been shown to influence net use [7,19,20], and are likely to interact with individual, household, community and environmental factors in complex ways to determine attitudes towards net use and the feasibility of net use for a given individual or household.

A recent review of literature on determinants of net use highlights the need for greater understanding of these determinants and the relationships between them [29]. In addition, net use among those who own nets is commonly interrupted by temporary, periodic or infrequent conditions, which can inhibit net use even among regular net users. These conditions include travelling, night work, sleeping in the fields during planting or harvest seasons or while tending livestock, attending late-night social events, disruption of usual sleeping arrangements, net unavailability due to washing or dirtiness, extreme fatigue, labour pains, illness or forgetfulness [13,27,30-32].

This study builds on a previous analysis at the net level [6] of the results of two sequential surveys in Ethiopia [3] that demonstrated certain modifiable factors concerning net use, such as improved net care and replacement. In the previous study, the outcome was use or non-use for each net [6], whereas here factors associated with the outcome of individual use or non-use were investigated. The current study adjusts for both household and net level factors, including the important net age and condition characteristics identified previously [6]. In contrast to most previous studies, including some in Ethiopia [19,33,34], this analysis was not restricted to high risk groups (children U5 and pregnant women), but examined net use in all age groups including the 5-24 year old school-age children and young adults, women of reproductive age, and adults age 50 years and older. School-age children are a group generally least protected by insecticide-impregnated nets in Africa [35], including Ethiopia [9].

Since availability of a net in the household is a prerequisite for use, only those households owning at least one net are included in this analysis, to avoid biasing the results by lack of net availability in some households. Net density (i.e. number of nets per person in each household) is also used to account for differing household sizes and intra-household access to nets. The goal of this study is to identify under-served groups and modifiable factors that could be used to better target efforts to increase net use and, hence, assist in long-term reduction of malaria transmission in Ethiopia.

## Methods

### The study setting and surveys

The characteristics, survey design and sampling for the two surveys analysed here have been described previously [6]. Briefly, the analysis focused on the regional states of Amhara, Oromia and Southern Nations, Nationalities and Peoples' (SNNPR) of Ethiopia. For the baseline survey in 2006, a multi-stage cluster random sampling with probability proportional to population size was used to select 224 clusters with 25 households in each cluster. For the Malaria Indicator Survey (MIS) in 2007, a nationally representative sample was selected using a two-stage design stratified by three domains: areas below 1,500 m, rural areas between 1,500 m and 2,500 m, and urban areas between 1,500 m and 2,500 m. To enable comparisons with the baseline survey, only MIS 2007 clusters for three regional states, Amhara, Oromia and SNNPR, were included in the analysis presented here. This is referred to as the MIS 3R 2007 sub-sample and comprised 245 clusters defined as census enumeration areas (EA). A simple random sample of 25 households was selected from all mapped households in each EA, with altitude and location of each household recorded using personal digital assistants equipped with Global Positioning System capability.

### Outcome and risk factor measurement

The survey questionnaires were both based on the MIS Household Questionnaire modified for the local conditions to include socio-economic factors [36] as has been described previously [37]. The questionnaire was translated and conducted in Amharic language and pilot-tested in a non-survey cluster to determine the validity of the pre-coded answers. Interviews were conducted with the head of household, or another adult if the head of household was absent or unable to respond for any reason.

During the interviews, age and gender of all residents was recorded; and the number of rooms (2006) or sleeping spaces (2007) noted. The respondents were asked about mosquito nets in their household: presence, number, type and who used which nets the previous night. Both surveys used net rosters to associate individuals with specific observed nets.

Reports of recent (within the last 12 months) indoor residual spraying of the household with insecticide were recorded. At MIS 2007, women of reproductive age completed the malaria knowledge questionnaire which included questions assessing knowledge of the cause, symptoms, danger signs, and preventive measures of malaria.

### Statistical methods

**Figure 1:**
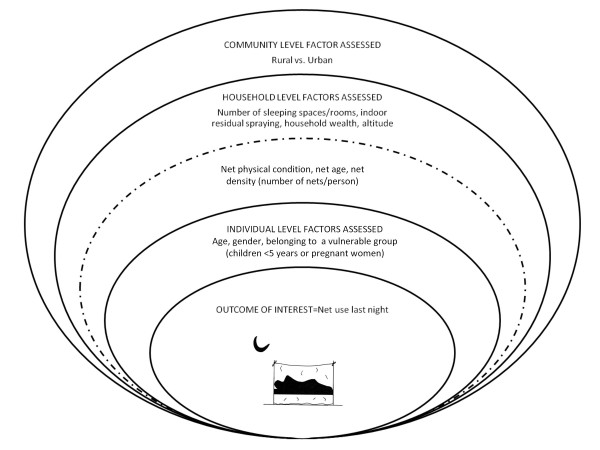
**Summary of data framework for analysis of association between individual use of net and explanatory factors**.

Figure 1 summarizes the framework for analysis of association between use of net and explanatory factors. The analysis was restricted to participants living in households owning at least one net. Each survey was considered separately. The reported use (or not) of a net by individuals the previous night was the dependent variable. Since this analysis was at the individual level, in order to examine characteristics of all nets in the household, including those not slept under, we developed summary variables for the age and condition of the nets in each household. Households with nets were classified as having 'none', 'some' or 'all' nets in good condition (i.e. a net without any holes). Proportion of nets older than one year was defined as households having 'none', 'some' or 'all' nets for 12 months or more. Net density was calculated by dividing the number of nets in a household by the number of people in the household.

The household wealth index was derived from relevant household characteristics using principal components analysis as previously described [38] and terciles defined (poorest, middle, richest). For the MIS 3R 2007, malaria knowledge score was derived based on methods previously described by Hwang *et al*. [15]. In brief, from the malaria knowledge questionnaire, a composite malaria knowledge score was calculated for each woman where every correct answer received a single point. The maximum knowledge score achieved out of 3,055 women was 18, but the median was 4 and 97% of women scored less than 10. Most households (2,392 of 2,701 or 89%) had only one woman respondent, 232 (10%) had two, 30 (1.1%) had three, 6 (0.2%) had four, and 1 (0.04%) had five respondents. To account for households that had more than one woman completing the malaria knowledge questionnaire (11% of 2,701 households), a mean malaria knowledge score was generated for every household and categorized into terciles (0-1, 2-4, and ≥ 5). School attendance was only obtained for women of reproductive age; school attendance was classified for a household as 'Yes' if any woman in the household had attended school.

Statistical analysis was conducted using Stata 8.2 (Stata Corporation, College Station, Texas). Descriptive statistics were used to examine the characteristics of the sample, and prevalence of outcomes and explanatory factors. To account for differences in the sampling design, prevalence estimates were adjusted for sampling weights. To investigate the association of reported net use by individuals the previous night and explanatory factors, hierarchical regression models were developed using generalized linear latent and mixed models (GLLAMM) [39]. The multi-level structure of GLLAMM allowed for non-independence of the household variables, enabled clustering of net observations within households and clusters, and allowed for variability at household and cluster levels. Univariate analysis was conducted for each potential explanatory factor. Multi-variable models were then developed by stepwise regression analysis for model selection. This involved starting with a null model then proceeding in a sequential fashion of adding/deleting explanatory variables if they satisfied the entry/removal criterion, which was set at 5% significance level using a log-likelihood ratio test. Since not all MIS 3R households had a malaria knowledge score, two multi-variable models were fitted: the first assessed independent risk factors in all eligible participants and the second assessed effects of women's malaria knowledge and school attendance adjusting for variables found to be independent risk factors in the first model. To investigate effect modification between malaria knowledge and school attendance previously described by Hwang *et al*. [15] an interaction term was included in the multivariable model.

### Ethical considerations

The protocols received ethical clearance from the Emory University Institutional Review Board (IRB#1816 and 6389), the US Centers for Disease Control and Prevention ethical review committee (IRB#990132) and the Ethiopian Science and Technology Agency. For both surveys, informed consent to participate in interviews was sought from the heads of household in accordance with the tenets of the Declaration of Helsinki.

## Results

### Characteristics of the sample

**Figure 2:**
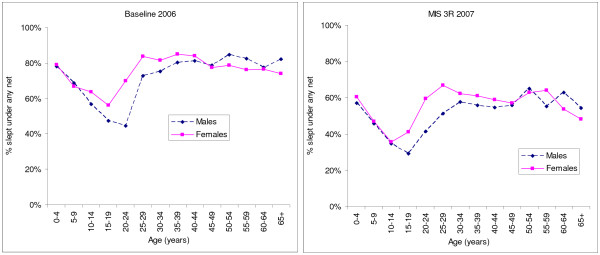
**Proportion of participants using nets the previous night by age and gender**.

The characteristics of the sample are summarized in Table 1 and Figure 2. A total of 12,678 participants in 2,468 households owning nets were included in the baseline 2006 analysis, while the MIS 3R 2007 analysis comprised 14,663 participants in 3,353 households owning nets. At baseline 2006 survey, 37.0% of households owned at least one net, while in MIS 3R 2007, the household net ownership had increased to 56.7%. At baseline 2006 survey, 59.4% of the nets were LLINs, while in MIS 3R 2007 LLINs comprised 95.1% of the nets. Despite an increase in the proportion of households owning at least one net, a lower proportion of participants (50.9%) reported using nets the previous night during MIS 3R 2007 compared to baseline 2006 (70.8%) among those people who had access to a net (Table 1). Figure 2 shows the proportions of net use by age and gender. For both surveys, there was a lower proportion of net use among people aged 5-24 years compared to the other age groups.

**Table 1:** Characteristics of sample population

Characteristics	Baseline 2006				MIS 3R 2007			
	
	Amhara	Oromia	SNNP	Total	Amhara	Oromia	SNNP	Total
Number of clusters	160	32	32	224	108	97	40	245

Number of HHs surveyed	4,101	809	798	5,708	2,609	2,321	980	5,910

Number of HHs owning nets	1,688	366	414	2,468	1940	960	453	3,353

Proportion of HHs owning at least one net (%)	34.7	45.4	51.2	37.0	74.4	41.4	46.2	56.7

Number of participants	19,059	4,428	4,397	27,884	10,733	10,266	4,082	25,081

Number of participants in HHs owning nets	8,298	2,019	2,361	12,678	8,381	4,342	1,940	14,663

Proportion of participants using net last night:*								

All ages	70.2	76.9	65.2	70.8	54.2	48.8	49.4	50.9

Children U5	77.9	81.9	75.1	78.4	63.4	56.4	56.2	58.7

Children age 5-14 years	63.8	74.6	51.3	64.2	46.1	41.5	34.2	41.7

Women age 15-49 years	75.3	78.8	71.7	75.5	62.7	55.5	55.1	58.1

Pregnant women	80.3	83.5	82.8	81.2	62.7	70.4	63.1	66.1

Persons age ≥ 50 years	78.8	81.7	81.4	79.4	59.7	53.7	69.2	59.1

Net density (average nets per person) in HHs owning nets	0.36	0.30	0.26	0.34	0.45	0.39	0.42	0.43

**Net condition in HHs owning nets, assessed by proportion of HH with:**								

No good nets (0%)	3.4	33.6	14.0	9.7	19.3	34.9	30.0	25.3

Some good nets (1-99%)	3.2	4.4	3.4	3.4	11.7	9.3	9.1	10.7

All good nets (100%)	93.4	62.0	82.6	86.9	68.9	55.8	60.9	64.1

**Net age in HH owning nets, assessed by proportion of HH with:**								

No nets > 12 m old (0%)	88.2	83.3	88.7	87.6	76.5	73.5	89.9	77.5

Some nets > 12 m old (1-99%)	3.4	0.6	0.7	2.6	7.6	3.1	6.0	6.1

All nets > 12 m old (100%)	8.4	16.1	10.6	9.9	15.8	23.4	4.2	16.4

*Among those people living in a household with ≥ 1 net

*HHs *households; *LLIN *long-lasting insecticidal net; *m *months

### Associations between net use and explanatory factors: Baseline survey 2006

Univariate logistic regression analysis of the associations between net use at baseline 2006 and explanatory factors is shown in Table 2. Factors associated with increased net use among participants were: age 25-49 years (OR = 1.5; 95% confidence interval [CI] 1.2-1.8) or age 50 years and above (OR = 1.3, 95% CI 1.0-1.7) compared to children U5; female gender (OR = 1.3; 95% CI 1.2-1.5); children U5 compared to other ages (OR = 2.0; 95% CI 1.7-2.4); pregnant women compared to other participants (OR = 3.8; 95% CI 2.3-6.3); increasing proportion of good nets in the household (Ptrend = 0.004); and increasing net density (Ptrend < 0.001). Reduced net use among participants was associated with: age 5-24 years compared to children U5 (OR = 0.2; 95% CI 0.2-0.3) (see Figure 2); increasing proportion of nets older than one year (Ptrend < 0.001); and increasing household altitude (OR_[household altitude > 2000 m] _= 0.1; 95% CI 0.03-0.1).

**Table 2:** Baseline 2006: univariable logistic regression analysis of association between individual use of net and explanatory factors in households owning at least one net

Factors		Total persons N = 12,678	Persons used net last night N = 8,945	% of persons using net	Odds Ratio	95% CI	*p-value*	*p*-value test for trend (> 2 categories)
***Individual characteristics***								

Age group (years)	< 5	2,073	1,621	78.2	1.0			
		
	5-24	6,081	3,680	60.5	0.2	0.2-0.3	< 0.001	
		
	25-49	3,432	2,781	81.0	1.5	1.2-1.8	< 0.001	
		
	50+	1,092	863	79.0	1.3	1.0-1.7	0.042	

Gender	Male	6,271	4,308	68.7	1.0			
	
	Female	6,407	4,637	72.4	1.3	1.2-1.5	< 0.001	

Child U5	No	10,605	7,324	69.1	1.0			
	
	Yes	2,073	1,621	78.2	2.0	1.7-2.4	< 0.001	

Pregnant woman	No	12,446	8,751	70.3	1.0			
	
	Yes	232	194	83.6	3.8	2.3-6.3	< 0.001	

***Net characteristics***								

Proportion of good nets (in HH with nets)	None (0%)	1,213	745	61.4	1.0			Ptrend = 0.004
		
	Some (1-99%)	535	421	78.7	2.5	1.5-4.01	< 0.001	
		
	All (100%)	10,930	7,779	71.2	1.6	1.2-2.0	0.001	

Proportion of nets older than 1 year (in HH with nets)	None (0%)	11,025	8,073	73.2	1.0			Ptrend < 0.001
		
	Some (1-99%)	349	277	79.4	1.1	0.8-2.2	0.326	
		
	All (100%)	1,304	595	45.6	0.1	0.04-0.1	< 0.001	

***Household characteristics***								

Number of rooms (per additional room)					0.9	0.8-1.0	0.182	

Net density	< 0.5	11,139	7566	67.9	1.0			Ptrend < 0.001
		
	≥ 0.5 < 1.0	1,437	1287	89.6	5.1	3.5-7.5	< 0.001	
		
	≥ 1.0	102	92	90.2	9.0	2.8-28.6	< 0.001	

Indoor residual spraying	Not sprayed	9,507	6452	67.9	1.0			
	
	Sprayed < 12 m ago	3,171	2493	78.6	0.9	0.7-1.1	0.375	

Wealth index quintiles	Poorest	4,291	3,107	72.4	1.0			Ptrend = 0.282
		
	Middle	3,919	2,840	72.5	0.9	0.7-1.1	0.332	
		
	Richest	4,468	2,998	67.1	0.9	0.7-1.1	0.27	

Altitude	< 1000 m	2,597	1,889	72.7	1.0			Ptrend = 0.796
		
	≥ 1000-2000 m	7,604	5,274	69.4	0.7	0.6-0.9	0.015	
		
	> 2000 m	2,477	1,782	71.9	0.1	0.03-0.1	< 0.001	

Table 3 shows the multi-variable associations between individual net use at baseline 2006 and explanatory factors. Factors independently associated with increased net use were: age 25 to 49 years (OR = 1.4; 95% CI 1.2-1.7) compared to children U5; female gender (OR = 1.4; 95% CI 1.2-1.5); increasing proportion of good nets (with no holes) in household (Ptrend = 0.002); and increasing net density (Ptrend < 0.001). Reduced net use by participants was independently associated with age 5-24 years (OR = 0.2; 95% CI 0.2-0.3) compared to children U5.

**Table 3:** Baseline 2006: multi-variable logistic regression analysis of association between individual use of net and explanatory factors in households owning at least one net

Risk factors		Odds Ratio	95% CI	*p-value*	*p*-value test for trend (> 2 categories)
Age (years)	5-24	0.2	0.2-0.3	< 0.001	
	
	25-49	1.4	1.2-1.7	< 0.001	
	
	50+	1.3	1.0-1.6	0.101	

Gender (Female)		1.4	1.2-1.5	< 0.001	

Proportion of good nets in HH	Some (1-99%)	4.6	2.5-8.3	0.01	Ptrend = 0.002
	
	All (100%)	1.5	1.1-2.1	0.033	

Net density	≥ 0.5 < 1.0	5.8	3.9-8.7	< 0.001	Ptrend < 0.001
	
	≥ 1.0	6.0	1.9-19.5	0.003	

### Associations between net use and explanatory factors: MIS 3R 2007 survey

Table 4 summarizes the univariate logistic regression analysis of the associations between net use at MIS 2007 and explanatory factors. Factors associated with increased net use among participants were: female gender (OR = 1.3; 95% CI 1.2-1.5); children U5 compared to other ages (OR = 1.8; 95% CI 1.5-2.3); pregnant women compared to other participants (OR = 3.0; 95% CI 2.0-4.5); increasing proportion of good nets in the household (Ptrend = 0.042); increasing net density (Ptrend < 0.001); increasing malaria knowledge (Ptrend = 0.004); and urban clusters compared to rural clusters (OR = 2.0; 95% CI 1.3-3.1). Reduced net use among participants was associated with: age 5-24 years (OR = 0.3; 95% CI 0.2-0.3) compared to children U5 (see Figure 2) all nets in households older than one year (OR = 0.3; 95% CI 0.1-0.6); increased number of sleeping spaces (OR = 0.5; 95% CI 0.5-0.6); and increasing household altitude (Ptrend < 0.001).

**Table 4:** MIS 3R 2007*: univariable logistic regression analysis of association between individual use of net and explanatory factors in households owning at least one net

Factors		Total persons N = 14,663	Persons used net last night N = 7,354	% of persons using net	Odds Ratio	95% CI	*p-value*	*p*-value test for trend (> 2 categories)
***Individual characteristics***								

Age group (years)	< 5	2,323	1,329	57.2	1.0			
		
	5-24	6,932	2,959	42.7	0.3	0.2-0.3	< 0.001	
		
	25-49	3,902	2,249	57.6	1.1	0.8-1.4	0.604	
		
	50+	1,506	817	54.2	1.2	0.8-2.0	0.4	

Gender	Male	7,264	3,455	47.6	1.0			
	
	Female	7,399	3,899	52.7	1.3	1.1-1.5	< 0.001	

Child U5	No	12,340	6,025	48.8	1.0			
	
	Yes	2,323	1,329	57.2	1.8	1.5-2.3	< 0.001	

Pregnant woman	No	14,423	7,213	50.0	1.0			
	
	Yes	240	141	58.8	3.0	2.0-4.5	< 0.001	

***Net characteristics***								

Proportion of good nets (in HH with nets)	None (0%)	3,791	1529	40.3	1.0			Ptrend = 0.042
		
	Some (1-99%)	1,879	1016	54.1	2.5	1.8-3.5	< 0.001	
		
	All (100%)	8,993	4809	53.5	1.8	1.3-2.6	< 0.001	

Proportion of nets older than 1 yr (in HH with nets)	None (0%)	11,104	5885	53.0	1.0			Ptrend = 0.091
		
	Some (1-99%)	1,055	556	52.7	1.1	0.3-4.2	0.939	
		
	All (100%)	2,504	913	36.5	0.3	0.1-0.6	0.002	

***Household characteristics***								

Number of sleeping spaces (per additional space)					0.5	0.5-0.6	< 0.001	

Net density	< 0.5	11,040	5040	45.7	1.0			Ptrend < 0.001
		
	≥ 0.5 < 1.0	3,224	2048	63.5	2.6	2.0-3.4	< 0.001	
		
	≥ 1.0	399	266	66.7	3.9	2.1-7.3	< 0.001	

Indoor residual spraying	Not sprayed	11,790	5749	48.8	1.0			
	
	Sprayed < 12 m ago	2,873	1605	55.9	1.4	1.0-2.1	0.079	

Wealth index quintiles	Poorest	4,912	2,415	49.2	1.0			Ptrend = 0.384
		
	Middle	4,880	2,424	49.7	1.0	0.5-1.7	0.868	
		
	Richest	4,871	2,515	51.6	0.7	0.5-1.0	0.071	

School attendance*	No	9,857	4,891	49.6	1.0			
	
	Yes	2,946	1,556	52.8	0.9	0.7-1.2	0.63	

Malaria knowledge mean score (terciles)*	0-1	3,251	1,517	46.7	1.0			Ptrend = 0.004
		
	2-4	4,758	2,438	49.3	2.3	1.3-4.2	0.005	
		
	≥ 4	4,794	2,582	53.9	2.1	1.0-4.0	0.036	

Altitude	< 1000 m	416	201	48.3	1.0			Ptrend < 0.001
		
	≥ 1000- ≤ 2000 m	9,996	5211	52.1	0.5	0.4-0.8	0.006	
		
	> 2000 m	4,251	1942	45.7	0.3	0.2-0.4	< 0.001	

***Cluster characteristics***								

Location of cluster	Rural	12,060	5981	49.6	1.0			
	
	Urban	2,603	1373	52.7	2.0	1.3-3.1	0.001	

* Household women's knowledge score and school attendance available for 12,803 participants in 2,701 households owning nets where women of reproductive age completed knowledge questionnaire

Multi-variable associations between net use at MIS 3R 2007 and explanatory factors are shown in Table 5. Since not all households had a malaria knowledge score, two multi-variable models were fitted: the first assessed independent risk factors in all eligible participants (n = 14,663); and the second assessed effects of malaria knowledge and school attendance (n = 12,803) adjusting for variables found to be independent risk factors in the first model. Restriction of the sample size in Model II did not change the variables included in the multivariate model.

**Table 5:** MIS 3R 2007: Multi-variable logistic regression analysis of association between individual use of net and explanatory factors

Model*	Risk factors		Odds Ratio	95% CI	*p-value*	*p*-value test for trend (> 2 categories)
I	Age (years)	5-24	0.3	0.2-0.4	< 0.001	
	
		25-49	1.1	0.8-1.5	0.474	
	
		50+	1.2	0.7-2.0	0.415	
	
	Gender (Female)		1.3	1.1-1.6	0.001	
	
	Proportion of good nets in household	Some (1-99%)	1.8	1.2-2.8	0.006	
			
		All (100%)	1.6	1.2-2.1	0.003	
	
	Proportion of nets older than 12 m	Some (1-99%)	0.9	0.5-1.6	0.781	
			
		All (100%)	0.5	0.3-0.7	< 0.001	
	
	Number of sleeping spaces in HH (per additional space)		0.6	0.5-0.7	< 0.001	
	
	Net density	≥ 0.5 < 1.0	2.0	1.5-2.7	< 0.001	Ptrend = 0.001
			
		≥ 1.0	2.0	0.9-4.2	0.077	
	
	Altitude	≥ 1000- ≤ 2000 m	1.4	0.9-2.4	0.142	Ptrend < 0.001
			
		> 2000 m	0.5	0.3-1.0	0.068	
	
	Urban clusters		2.5	1.5-4.1	< 0.001	

II**	Malaria knowledge mean score (terciles)	2-4	1.6	1.2-2.2	< 0.001	Ptrend < 0.001
			
		≥ 5	2.4	1.4-2.4	< 0.001	
	
	School attendance	Yes	1.1	0.8-1.5	0.719	

*Model I assessed independent risk factors in all 14,663 participants (in 3,353 households owning nets). Model II assessed effect of household women's knowledge and school attendance in 12,803 participants (in 2,701 of these households where women of reproductive age completed malaria knowledge questionnaire) adjusting for the effects of independent risk factors from model I.

**Test for interaction between malaria knowledge and school attendance did not reveal any statistically significant effects (Wald Test p-value = 0.781)

Factors independently associated with increased net use were: female gender (OR = 1.3; 95% CI 1.1-1.6); increasing proportion of good nets in household (OR_[all nets in HH good] _= 1.6; 95% CI 1.2-2.1); increasing net density (Ptrend < 0.001); women's malaria knowledge (Ptrend < 0.001); and urban clusters (OR = 2.5; 95% CI 1.5-4.1). Reduced net use by participants was independently associated with: age 5-24 years (OR = 0.3; 95% CI 0.2-0.4) compared to children U5; number of sleeping spaces (OR _[per additional space] _= 0.6, 95% CI 0.5-0.7; increasing proportion of nets older than one year (OR _[all nets older than 12 months] _= 0.5; 95% CI 0.3-0.7); and increasing household altitude (Ptrend < 0.001). Test for interaction between malaria knowledge mean score and school attendance did not reveal any statistically significant effects (Wald test p-value = 0.781)

## Discussion and Conclusion

This paper describes determinants of net use as assessed in two consecutive household surveys in 2006 and 2007 in the three largest regional states of Ethiopia. Decline in net use (in households owning nets) between these surveys had been observed, despite increase in overall net ownership [3]. A previous study examined use in a net level analysis (i.e. whether or not each net had been used the previous night) [6], and demonstrated that increasing net age and increasing damage of nets were both associated with a lower likelihood of nets being used. In that study, increased net density was associated with decreased likelihood of a net being used, which is logical since the more nets there are in a household (especially above a density of one net per person), the less the chance of each one being used.

Most previous studies (with some exceptions [19,40]) have expressed net use not as a proportion of nets used, but as a proportion of persons (usually children U5 or pregnant women) using nets [33,34,37,41]. This individual outcome of net use is used in the current study, but includes all individuals in the sampled households since the programme goal is universal coverage. GLLAMM was used to account for both household characteristics and the previously identified net level determinants of use. Using individual net use as an outcome, it would be expected that increased net density would be associated with increased net use, as we found here in both 2006 and 2007. Net density by household was classified in three categories as < 0.5, 0.5 - < 1 and ≥ 1 nets per person, and net use was found to be five-fold higher (in 2006) or two-fold higher (in 2007) when net density was greater than 0.5. This suggests that a net distribution target higher than one net per two persons (such as two nets per three persons or if possible one net per person) is more likely to maximize net use, especially if there are sufficient nets to cover all sleeping spaces. While it is commonly stated that lack of access to sufficient nets within households may be an explanation for low use, there are very few other studies that examined net use in relation to household net density while adjusting for other factors [8].

This study confirmed the previously demonstrated impact of the two related factors of net age and condition on likelihood of net use. In 2006, net use was about twice as likely if some or all the nets in the household were undamaged, while in 2007 net use was one third as likely if all the nets in the household were older than 12 months. The frequent observation of nets in poor condition has been previously reported from Ethiopia [42] and nets owned for less than 12 months were significantly more likely to be used [19]. These findings have implications for care and replacement of nets. The programme should promote net repair and maintenance and also conduct educational activities to change the perception that nets with a few holes are no longer worth using.

Overall, females in both surveys slept under a net the previous night 1.3-1.4 times as often as males. Less than 2% of the population reported being pregnant at each survey. In univariate analysis, pregnant women were at least three times more likely than the general population to use nets, but this factor did not remain associated with net use in the multi-variate model. Net use by age group showed surprisingly that children U5 are not the group with highest net use: persons older than 24 years were more likely to use nets in both surveys, although the association was only statistically significant for the 25-49 year old age group in the 2006 survey. It is clear that children and young adults age 5-24 years use nets much less frequently than children U5 or persons over 24 years (Figure 2). Less frequent use by school-age children has been observed in many other African counties [35] and it would be interesting to conduct a study of net use in which children's own school attendance, access to nets, and knowledge are taken into account, rather than using proxies from women in their households. It is critical that more attention is paid to increasing net use in children, teenagers and young adults (e.g. perhaps by school-based education campaigns and activities), since they likely contribute significantly to transmission and are at risk of severe disease, if infected.

The finding of higher net use in urban compared to rural areas in MIS 2007 was surprising, since urban dwellers might be expected to associate net use with villagers and rural life. However, another study in Ethiopia [19] observed the same higher use of LLIN in urban areas. Possible reasons include: higher levels of education; potentially larger and more diverse social networks perhaps due to greater population density; or a more progressive attitude among urban dwellers leading to earlier adoption of unfamiliar strategies. Other potential unmeasured factors (other than those for which we adjusted such as net density, socio-economic status, malaria knowledge, and IRS status) should be explored further.

Women's malaria knowledge and school attendance were assessed only in the MIS 3R 2007 survey, and expressed as terciles of an index of correct scores, applied at the household level. The results shown here support those of Hwang *et al*. [15] and others [33,34] that increased women's knowledge of malaria can improve net use by individuals and/or members of their household. However, the interaction effect previously described [15], whereby higher women's knowledge (score of > = 1) only increased net use in themselves and their children U5 if the women had not attended school, was not observed here. This may be due to the different outcome (net use in all ages), different quantification of knowledge scores (in terciles), or application of women's knowledge scores and school attendance at the household level.

Potential limitations of this study include the fact that a number of other possible determinants of net use were not assessed, including, but not limited to, perceptions of malaria risk, the proportion of nets that were hanging, and the opinions or beliefs of householders about their ability to hang nets and/or difficulty of hanging nets in their homes. In addition, aspects of net condition other than the presence of holes (e.g. cleanliness, smell and perceived effectiveness of insecticide) that might affect a person's choice to sleep under a net were not measured. It may be that once the novelty of newly distributed nets has worn off, people stop using nets because the practice grows tiresome and their risk perception returns to initial lower levels, suggesting the need for sustained behaviour change communication. Differences in climate factors or seasonality between the two surveys are potential limitations, but a previous study examined this question extensively [3] and showed that slight differences in survey timing in each year did not result in important differences in rainfall or temperature conditions between the surveys. Differences in sampling strategy between surveys were also examined and were found to be highly unlikely to have introduced bias in the estimate of net use [3].

Examining net use from both the net level [6] and the individual level (present study) while restricting the data to only households owning nets enables adjustment for the different levels of analysis (net, household, cluster) and avoidance of spurious associations, while using net density in the models allows accounting for changes in net ownership over time. Increasing availability of nets within households (i.e. increasing net density), and improving net condition while focusing on education and promotion of net use, especially in school-age children and young adults in rural areas, are crucial areas for intervention to ensure maximum net use and consequent reduction of malaria transmission.

Despite large distribution of new nets between the surveys, the pool of nets in households in 2007 was older and in less good condition than 2006 due to nets remaining from earlier distributions as well as rapid accumulation of damage to nets [43]. Older nets in poor condition are used less than new nets [5]. The study raises important issues about the longevity of nets and when a net is considered 'expired' or no longer suitable for use. More research on this issue is warranted since firstly nets considered to be too damaged may not be used; and secondly the accumulation of 'expired' nets in households would inflate estimates of net ownership in standard household surveys and net density estimates as used in this analysis. Qualitative research investigating when households would consider nets unfit for use, and determining whether this expectation would be realistic - from an operational and programmatic perspective - is necessary. Why damaged and unused nets are kept, or how they are disposed of is also of interest. Such information would generate realistic assessments of average usable net life and improve planning of sensible and sustainable replacement and communication strategies instead of blanket mass distributions at predetermined intervals.

## Competing interests

The authors declare that they have no competing interests.

## Authors' contributions

PG, JH, RR, FR, TG and PE conceived and planned the study; PG, JH, TG, AM, ES, ZT, AW and RR trained staff, carried out the survey and managed the data; JN and PG undertook data analysis with input from JH, AM, and RR; PG, JN and AP wrote the paper with input from JH, AM, RR, FR, TG, ZT and PE; all authors approved the final draft.
